# Robert Koch, malaria pioneer

**Published:** 2022-05-17

**Authors:** Jan Peter Verhave

**Affiliations:** 1 Retired from the Departments of Medical Microbiology and International Health, Radboud University Medical Centre Nijmegen, The Netherlands

## Abstract

The role of Robert Koch in the early discoveries of the malaria lifecycle and the complex of diseases, the development of immunity, quinine prophylaxis and the mosquito theory has fallen into oblivion. As a mature and famous hygienist, Koch had travelled the Old World, where malaria was endemic. His first studies took place in Tanganyika, German East Africa (now Tanzania) in 1898 and thereafter in Italy and the East Asian archipelago. As malaria in Germany did not offer a sufficiently endemic situation, he chose the Istrian island of Brioni (Kroatia) to eliminate malaria. Because virtually all of Koch’s publications are in German, his achievements on malaria never settled in the common memory of tropical medicine. Around the turn of the century the race to elucidate the transmission pathway through mosquitoes took place and though he hardly yielded any honour of priority, his research certainly determined the speed by which British and Italian contenders made their ways. His exertion and interference led to the awarding of the 2^nd^ Nobel Prize in Medicine to Ronald Ross only, leaving Giovanni Battista Grassi to draw the blank. Proof of this intervention in the otherwise well-known quarrel at the start of modern malaria research shows once more how personal characters may clash or join forces.

## Introduction & Justification

In the early 1970s, I did research on the immune responses against developmental stages of malaria parasites. My PhD work would form the basis for present human vaccine trials [1], and after completing my thesis I extended activities to West-Africa and served as a mentor for PhD students and their assisting Master students. After retirement, I wrote biographies of the Dutch malariologist Nicolaas Swellengrebel and the American bacteriologist and science journalist Paul de Kruif [2]. My interest in the history of medical science led me to the present subject.

**Figure 1.**
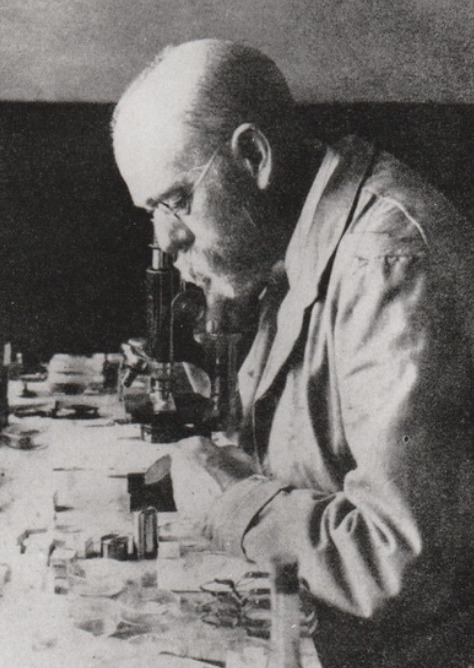
Robert Koch.

Among today’s malariologists the name of Robert Koch is no longer associated with his discoveries on malaria. Koch (1843-1910) was Director of the Imperial Health Department in Berlin, and had demonstrated the bacterial agents causing anthrax, tuberculosis and cholera. Because he was already famous for these discoveries, his role in the history of malaria has remained under-exposed. Koch’s reflections and publications in German, if at all accessible, are somewhat lengthy to the taste of present-day readers and researchers. Yet, they contain a wealth of original thinking and scientific experiments that are worth to be known. To make his publications accessible, this account follows Koch (Figure 1) on his travels to several malaria-ridden countries, and selection from the reports on his laboratory and field studies are presented [3].

Most of Koch’s publications were collected in the *Gesammelte Werke von Robert Koch*, in 1912 (3 volumes; GW), and are now accessible online via the Robert Koch Institute, Berlin. Only once, an indepth study of part of Koch’s research on malaria was made by Markus Schnöpf, for his MA thesis, in German; it is not readily available in academic libraries [4].

By following Koch’s part in the discovery race of the transmission, control and treatment of malaria, his personal attitude towards the international forum of contenders may become understandable.

The first chapter is an abbreviated and freely translated version of Koch’s letters in his Travel Accounts concerning malaria in German East Africa (present day Tanzania) [5]. This condensed adaptation of Koch’s report (40 pages), in which I maintain the writer in the first person, serves as an introduction to the next chapters about Koch’s German Malaria-Expedition to Italy, the Indian archipelago and his control project in Istria. What Koch observed in East Africa, and thereafter, has long since become common knowledge. It is revealing to realise how precise and correct most of his descriptions and conclusions were, around the emergence of the mosquito-hypothesis and the early control attempts (i.e. by breaking the transmission cycle).

## Part I: Tropical Malaria in German East Africa

In December 1897 Koch arrived in German East Africa (Tanganyika) where he intended to study Texas fever and tsetse- or surra fever of cattle. Because malaria was still a parasitic disease that required a great deal of unravelling, Koch went on a hygienic mission to the Usambara mountains. The workers on the new railway from Dar es Salaam to Muheza suffered from malaria and his colleague Dr. Friedrich Plehn (1862-1904), who had studied malaria in Cameroon, recently settled in nearby Tanga and consulted Koch on the issue of the rapidly fatal malaria. Koch made his microscopic diagnoses among local and European patients in the Western Usambaras and at the agricultural station of Kwai.

Later he settled in Dar es Salaam and got his own laboratory at the Government Hospital (present day Ocean Road Hospital). Besides humans, he also found monkeys to be infected with malaria parasites. He was not impressed by the prevailing standard of medicine and promoted microscopical examination of (thin) blood smears, using the Romanovski staining which was modified by Dr. Gustav Giemsa (1867-1948), who served in Dar es Salaam from 1895-1898. He also met military surgeon Heinrich Ollwig (1863-1914), whom he invited as his assistant in malaria research. Koch’s stay in Ocean Road Hospital is commemorated by a brass plate, which mentions (in German) his fundamental researches in malaria [6]. Back in Berlin, on 20 May 1898, Koch had his reports published in his "Reise-Berichte", whilst Ollwig took the opportunity to follow a short course in parasitology in Hamburg. Soon, they would embark on an expedition to Italy and East Asia. Koch wrote about his studies in East-Africa:

**Travel-account from Dar-es-Salaam, 25 February 1898.**

*German East-Africa is renowned as unhealthy, solely because of malaria. None of the European infectious diseases, including tuberculosis, are important here, but malaria accounts for 54% of the hospitalized patients.*

**The types of malaria**

*It is exclusively the tropical form of malaria, with its quotidian fever attacks, which accounts for 90% of all malaria in this part of the world. Without treatment it leads to anaemia and chronic suffering with recrudescences. It is often fatal and called pernicious or blackwater fever. I examined 154 suspected patients, but using the criteria (clinical presentation, parasites in the blood and response to quinine) only 72 were confirmed. Of these 7 were of the tertian type and 1 of the quartan type.*

**The course of tropical malaria**

*I succeeded to follow patients without treatment, of whom I registered temperature and parasites 6 times per day. To my surprise I found that the tropical malaria is not at all so irregular as is stated. Fever bouts were not of the quotidian, but of the tertian type. They take longer than European tertian fever, almost 2 days (36 hours as compared to 48 in tertian) and drop in the morning of the second day, to start rising again in the evening.*

*The attacks may disappear without quinine intervention, and recrudesce later on.*

**The parasite**

**Figure 2.**
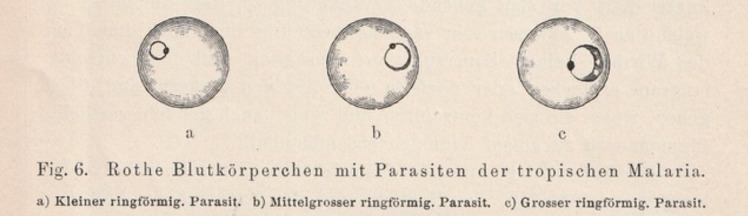
Parasites of "tropical malaria" in circulating red blood cells.

**Figure 3.**
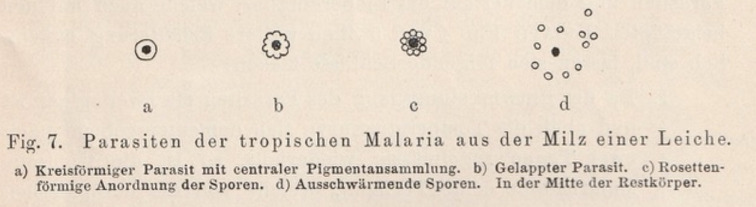
Maturing parasites with pigment in the spleen of a deceased patient.


*The agent of tropical malaria is quite similar to the description of quotidian malaria parasites in other tropical countries. It has a ring form with an intensively staining knob, and changes in size, a phenomenon essentially relating to the fever bouts (Figure 2). It has much less pigment than tertian or quartan parasites. I found mature stages with abundant pigment in spleens of patients who had died of malaria and explain these as an effect of lack of oxygen after death. The parasites also form spores, not in the blood but very nicely in the spleen; they look smaller, but much alike the parasites of other malaria types. Rosettes of 8-12 small balls around clumps of pigment, which end up in splitting off from the residual body (Figure 3).*

*Relating to the clinical course, small rings are seen in the blood during fever. They are at their maximum size towards the end of the fever, and in the two fatal cases I observed 10 and 50% of the red blood cells were infected, not rarely with two or more parasites. The parasites reach their maturity not in the peripheral blood, but in the spleen and other organs, where they form spores. Their dispersion coincides with the remounting of fever and the reappearance of small rings in the blood.*

*Quinine works optimally when given some hours before the beginning of the attack, thus when parasites are maximal in size. It only requires a microscope to determine accurately andeasily the right moment of treatment. A problem is the tendency of tropical malaria to recrudesce for about a month. I advise to take 1 g of quinine every 5 days for 1-1½ months.*

**Crescent forms**

*These bodies are enigmatic and generally considered "Dauerforms", which circulate without contributing to clinical symptoms. They were believed to give rise to new generations of parasites, which cause relapsing malaria. But I found them mostly for a longer period and associated with the end phase of disease. These patients had had several fever attacks, alternating with small and large rings. After the last bout of fever they disappeared, only to be replaced by the crescents, which remained circulating for 14 days in decreasing numbers. The change was not induced by quinine and the fever did not reappear. The patients, who remained under observation for a longer period, showed no recrudescences. I conclude that the parasite could no longer survive in the body, which had become immune.*

**Incubation time**

*It is often said, also by physicians, that patients can get malaria one or a few days after having been exposed on a hunting tour. This is impossible in my view, as the few acquired parasites have to accumulate in two-day cycles and thus need a longer period to cause the characteristic malaria fevers. I estimate this period to be 10-12 days and 5/7 generations. In this respect I was lucky to be told a story by the captain of the warship Condor: eight seamen had visited the coast of local Moa Bay and spent the night ashore. Six fell ill with typical malaria 11-13 days later, despite the use of quinine during the 2 days of the expedition.*

**The transmission**

*Proof of transmission is not available and one can only guess. Water may be contagious but the more I study malaria, the more I consider mosquitoes to be the only source of infection. Malaria and mosquitoes go together everywhere. I visited the coastal island of Chole, which used to be the (malaria-free) health resort of Arabs from Zanzibar; only there could I sleep without a mosquito net [7]. In the mainland mountains there is no malaria where there are no mosquitoes (i.e., above 1200 m). Mosquitoes and malaria fluctuate in a parallel way with the seasons (*Koch was not yet aware of the studies and results of Ronald Ross in British India, see note 12*).*

*But mostly, I was struck by the analogy with Texas fever and bloodsucking insects. It is not a direct transfer, but the parasites require a development inside the insect, pass to the eggs and larvae, and then again into the final host. Similarly, mosquitoes may pass the blood parasites to their offspring.*

*To prove this, one would need to do experiments with animals, but unfortunately, all my attempts at infection with human blood failed and though I identified blood parasites in many birds, reptiles, dogs and particularly monkeys, I could not find animals with human-like parasites. Malaria parasites are thus host-specific and I conclude with regret that an animal model for human parasites is not at hand.*

**Immunity**

*Some people and even whole populations ("Völkerschaften") in endemic areas remain free of malaria and must be more or less immune. I did everything I could to examine any black person from the coastal area with fever, but virtually never found malaria parasites. However, the people from the Usambara mountains are not immune. Neither are the imported labour people from China in Mohoro, or Indians in Dar es Salaam. Among the latter I studied a music band of 17 members, of whom 6 got tropical malaria! On the other hand, Indians who have settled for years along the East-African coast, remain quite healthy.*

*Thus, immunity is a reality that can be acquired. It is similar to what exists in Texas fever and I hope that for tropical malaria artificial immunisation can be achieved as well.*

**Treatment**

*Tropical malaria, even in severe and dangerous attacks, never causes cachectic aspects. It does not leave the patient with lasting changes and they recover completely, either spontaneously or after medication. Quinine is an efficient drug; 1 g suffices to clear the blood of parasites. Generally, one last fever bout follows ("night fever"), which is caused by the old generation of parasites, releasing its toxic metabolites in the blood. Important is the timing, best when the big rings appear. Fever is less reliable for malaria than the microscope. A malaria doctor without microscope and training will always grope his way in the dark [8].*

*Early treatment is important; several attacks disturb the stomach and quinine will not be absorbed. Quinine should be fresh (not spoiled) and the habit of wrapping it in paper to avoid the bitter taste, often results in passing the stomach without resorption.*

*Tropical malaria has the tendency of recrudescing (“Rückfälle”). Prophylaxis after treatment may prevent this. I have given three patients 0.5 g every third morning after treatment and they came down with fever again. Even a daily dose could not prevent this. But 1 g every fifth day seems to work. These experiments have to be expanded in a methodological way, with sufficient numbers of patients. I take 0.5 g every third day myself and remained free of fever.*

**Prevention**

*Prophylaxis is recommended for immigrants, military and seamen who have to pass through or stay for some time in malarious areas. It can also help the inhabitants of these areas to bridge the dangerous periods of the year. Additional measures against malaria are the following.*

*Drinking water has to be boiled and mosquito nets have to be used properly, well-sealed and without holes. Marshes have to be dried and cultivated, water correctly disposed of and houses well-constructed to offer families a healthy shelter. I am a public health man (“Hygieniker”) and it counts also for malaria that prevention is preferable over treatment.*

**Tertian malaria**

*The African form is not distinguishable from the European tertians [9]. I observed double tertian, which without microscope could have been mistaken for quotidian tropical malaria. It was caused by two parasite generations. I also observed tertian malaria to follow tropical malaria; it occurred to me that they mutually exclude each other, rather than coexisting. Treatment with 1-2 g of quinine is sufficient.*

**Blackwater fever (2 March 1898)**

*This is the most threatening disease for Europeans in German East Africa and has symptoms in common with malaria. Obvious is the blood in the urine, and patients weaken. I studied 16 patients with a mortality of 19%. In the blood of only two patients I found malaria parasites and no bacteria could be cultured. Patients developing blackwater fever were treated with quinine upon the demonstration of tropical or tertian parasites and with every dose a new attack of haemoglobinuria resulted. They must have developed sensitivity to quinine. All the other patients had no parasites and thus a direct relationship with malaria is not evident. It is merely quinine intoxication. People in German East Africa take quinine in an irresponsible way, with any unwell being or fever, and in high doses.*

**The Usambara mountains (5 March 1898)**

*I visited west Usambara to find out if the area was suitable for German immigrants to settle as farmers and for the establishment of a sanatorium. From Tanga on the coast it took a march of one week through Bondei land, via the Rufu river and the feared papyrus marshes of Tarawanda to Mkomasi and Mombo, up the mountains to the trial plantation of Kwai. The nearby Trappist friars in the mission post of Gare suffered from so-called acclimatisation fever and several had died. I found out that it was plain tropical malaria, which the friars had brought from the coast. I found no other cases of malaria in the area, and the locally born children from German missionaries are in rude health, so I presume that above 1200 m the mountains are free of malaria and mosquitoes. At 800 m I found cases of tertian malaria, and further down also tropical malaria. Of the thirty porters not a single one fell sick, but they were all men from the coast. When the autochthonous mountain people go down they may get malaria, which lasts for a couple of months and occasionally, may end fatally. If the mountain dwellers have no immunity to malaria, which seems to be the case, it follows that they find no opportunity in the mountains to acquire immunity the natural way; in other words, malaria is not present in the mountains. The natives are convinced that they get this illness because of the biting mosquitoes in the lowlands. The local word for mosquito as well as for fever is “Mbu"[10].*

Back in Germany, Koch lectured on 9 June, 1898 for the Deutsche Kolonial-Gesellschaft, Berlin-Charlot-tenburg, with the subject: “Observations of a medical doctor in the tropics”. A great number of academicians were present in gala uniforms and "Deutschland über Alles" was intoned; the German newspapers and medical journals reported the event with so much pride, that it initially made the Dutch Medical Journal to bring the news that Koch had discovered mosquitoes as transmitters of malaria. It also provoked a very irky reaction at the British side [11].

To the defence of Koch one may consider that the Berlin circle of the German Colonial Society did not consist exclusively of medical men; many dealt with exploration and geography, trade, settlement and mission. Among medical colleagues he might have selected his wordings more carefully. Newspaper journalists probably exaggerated the news.

The observations of Koch on malaria and its agents in East Africa are remarkable. His analysis of the parasite that causes “tropical fever” (to be called *Plasmodium falciparum*), its importance for diagnosis, its mature forms withdrawing from the circulation and the relation with fever bouts, the crescent forms and the implications for transmission were unique at the time, as were his assumptions on development of immunity.

While preparing for his next Malaria Expedition to the Indian Archipelago, Koch was informed about the discovery of Ronald Ross (1857-1932) in British India: malaria parasites develop inside mosquitoes and are transmitted through bloodsucking [12]. Koch wanted to confirm the discovery and proposed a pre-expedition to Italy where malaria was common. Early August 1898 he left for Rome and succeeded to confirm Ross' experiments, thus contributing to the mosquito theory as a scientifically based fact.

Ross and his mentor Patrick Manson (1844-1922) frequently exchanged letters. Manson wrote to Ross on 5 August, 1898 on the famous speech of Koch in Berlin [13]:

*It is the biggest piece of blatant egoism I have come across.*

Realising that Koch was a major contender, Manson to Ross, 2 September, 1898:

*Koch has gone to Rome to study malaria. Pity he didn't go there before. I sent him one of your reports... I also learned officially that Koch and a commission are going out to India, Africa and New Guinea for 2 years to study malaria. I hope you will scoop up all the cream before they get at the milking. Doubtless your mosquito discoveries have instigated this move. In his later lucubrations, Koch claims the mosquito theory as his own and never says a word about your work. Cool, is it not?*

Ross to Manson, 6 September, 1898:

*I see by the BMJ for August 20th p. 498, that Koch has gone to Italy to study malaria. It is quite possible that he will at once succeed in infecting men by the mosquito if he is lucky…*

## Part II: Two Visits to Italy

After his intensive encounter with malaria in German East Africa and on his way to Berlin, Koch made a short stop in Rome, where he met his colleague Giovanni Battista Grassi (1854-1925). This famous zoologist was also interested in the mosquito theory and Koch informed him that he intended to perform transmission studies. After the successful presentation of his African experience in Berlin, Koch was invited in 1898 by the Italian government to study the rampant malaria in that country. At the time there was considerable confusion about forms and species of malaria parasites in moderate climates and the tropics, and also the mode of transmission was a hot item. Koch was preparing for a major scientific expedition on malaria to the Far East and considered this invitation to Italy a welcome chance for a “wissenschaftlichen Vor-Expedition” [14].

The main objects of this preparative study travel were:

1. To give a clear picture about the various forms of malaria in Italy
2. The relation between Italian summer-autumn fevers, “febbri estivoautunnali” and tropical fever
3. To collect information about the aetiology, in relation to transmission by bloodsucking insects

In this chapter a collection of Koch’s statements from his own reports are given [15], as well as fragments of letters cited in the biography of Koch, by B. Möllers [16] (indicated as BMpag). I glued the citations into a readable narrative.

Koch set off on 11 August, 1898 to Milan, together with Richard Pfeiffer (1858-1945) and Hermann Kossel (1864-1925), both experts on blood protozoa. Being a celebrity, Koch arrived in Milan with a lot of fanfare and the local journals wrote that he would solve the malaria problem [17]. The Italian government charged Prof. Bartolomeo Gosio (18631944), director of the Laboratory for Hygiene in Rome, to assist him. Grassi had left the laboratory in Rome in July to study mosquitoes in the field. Koch:

*In several hospitals of Milan, Pavia, and especially in Rome, where we worked hard from 20 August to the end of September, we saw 120 patients: 32 with regular tertian malaria, 5 with quartan malaria, 78 with aestivo-autumnal malarial fever and 5 mixed infections. I was particularly interested in those cases with “summer-autumn malaria” and observed that they start as tertian fevers. This reminded me of the East-African malaria, which I had named tropical fever (“Tropenfieber”). The clinical signs did not differ very much and only the parasites were slightly bigger, and with more pigment, but not sufficient to decide upon different types. The difference is that I had not seen the daily and interrupted fevers in the African hospitals, apparently because the people there went more rapidly to a hospital. In Rome I saw few fresh cases, but mainly those that had been influenced by quinine and beginning immunity. After apparent cure the malaria parasites can show up again in the circulating blood and therewith the attacks, a recrudescence (“Recidiv”) of the disease. This may happen after weeks, months or years.*

*Based on reports from Italian researchers I had expected considerable differences, but I quickly found out that these only seemed so. The Italians study liquid blood without further manipulations, whilst I fix my smears and stain them, allowing me to observe more minute details. The aestivo-autumnal malaria and the tropical malaria are clearly caused by a single species of parasites. Both fevers are thus identical, and that is an important step for further studies. We now consider only three forms of human malaria parasites [18].*

*A special observation concerned the so-called “half-moon forms” and the flagellae (“Geiselkörper”) that originate from them. They were generally considered to be degenerate forms, because chromatin could not be seen. But thanks to the improved Romanowsky staining, we were able to demonstrate chromatin in the half moon parasites [19]. We saw that the flagellae directly came out of the chromatin bodies and propose the analogy with spermatozoa.*

*We also studied the human malaria of Rome in place and time. It occurred not in the city, but directly outside it was there abundantly. It must be the mosquitoes that are absent in the city, but as soon as the vegetation starts it teems with them. The fevers peak from June to the autumn (estivoautunnali). Something must have to happen in May that is essential for the sudden growth to 5-6 times the level of the months before. It would be highly important to unravel this factor.*

En passant, Koch had also treated some Roman patients suffering from blackwater fever with methylene blue, a chemical that might help when quinine was not supported. He stated that “it is not due to malaria but to a toxic effect produced by quinine” [20].

*Whilst I was preparing for the main expedition in Berlin, I learned about the first more exact news concerning the discovery of Dr. Ross in British India. It would be essential to gain full certainty about Ross’ ideas. These blood parasites of birds form an attractive analogy to study the aetiology of malaria, because they are extremely similar to human forms.*

*Together with Prof. Pfeiffer we demonstrated these parasites in goldfinches and sparrows, caught in the surroundings of Rome and we were lucky to quickly identify the right mosquito species. We could confirm the finding by Ross of Coccidia-like spheres in mosquitoes that had been fed blood with Proteosoma. We also closed a gap that Ross had left open: after fertilisation worm-like forms (“Wörmchen”) emerge in the stomach. The formation of these vermiculi was so easy to confirm, because I had already seen the origin of spermatozoa, the fertilisation process and the formation of these forms in Halteridium, in June 1898 together with Prof. Kossel. At that time, I was not aware of the investigations of MacCallum [21], but the priority is of course his.*

When Koch was preparing for his return to Berlin, Grassi returned to Rome from his fieldwork on mosquitoes, but the two did not meet. When he found out that Koch’s work was broadly discussed in Rome, he sent him his first publication on *Anopheles* mosquitoes. But Koch declared on a farewell party with medical doctors that he did not believe Grassi, because in Berlin the *Anopheles* did not cause malaria [22].

Back in Berlin, Koch continued his experiments with Proteosoma (later renamed *Plasmodium relictum*) in birds and mosquitoes (the model that Ross also used with chickens in Calcutta).

*16 November 1898 [23]: I work now exclusively on malaria, that is to say in the absence of human malaria with the related Proteosoma. I brought it*

**Figure 4.**
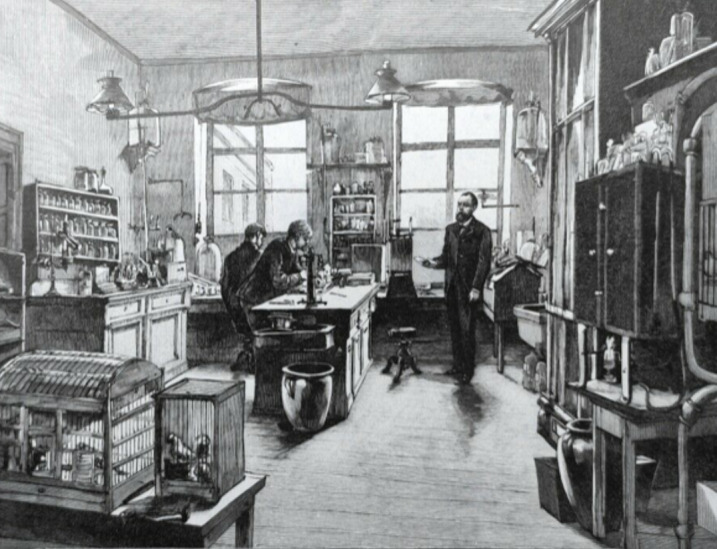
Koch in his Berlin laboratory; note the birdcages in the front-left.


*from Rome and maintain it by sub-inoculation. I rear the belonging mosquitoes in pure culture (“Reinkultur”). You have certainly read about the experiments with Proteosoma of Ross in Calcutta. Up till now I can fully confirm them. It is a highly miraculous developmental history: they multiply by division in the blood; but there is also a sexual development for the outside world (i.e., in an intermediate host). Male and female individuals are formed; the males form spermatozoa, fertilise the females and from there worm-like creatures emerge that move through the stomach wall of the mosquito and settle outside. Here they grow to ball-like forms, in which hundreds of sickle germs develop. That is how far I am; I don’t know yet what happens with these sickle germs.*

*19 November 1898: I am rather through with my studies on Proteosoma: The Coccidia-like balls on the stomach break at last and leave the germs free. The latter move into the salivary- or poison gland (“Speichel- oder Giftdrüse”) and must be inoculated by the sting of the mosquito. This would close the circulus vitiosus.*

*Following the procedures of Ross, we have tried to transfer the sickle germs back from mosquito to bird. On the 8th day after* Culex *had fed on infected canaries, we had them feed again on healthy birds, some of which got infected. Therewith, the whole cycle was described in accordance (“in Uebereinstimmung”) with Ross. Experiments with this so closely related parasite suggests an analogous development in human malaria and further research must one day lead to the corresponding forms. It saves a lot of uncertain and time-consuming probing, and leads the way for further malaria research. The so-called mosquito theory had ceased, at least for us, to be a theory; it had become a scientifically well-founded fact.*

Koch reported his findings to the “*Kaiserlichen Gesundheitsamt*”. Meanwhile in London, Patrick Manson wrote to his pupil in British India, on 30 October, 1898:

*I hear Koch has failed with the mosquito in Italy so you have time to grab the discovery for England.*

Koch’s fixation and staining of blood slides allowed him to conclude that aestivo-autumnal malaria in Italy is caused by the same parasite species as the tropical malaria in East Africa. He observed flagellation of crescent forms and supposed analogy with spermatozoa. He started experimenting with bird malaria and discovered ookinetes in mosquito guts, and back in Berlin, he confirmed the work of Ross.

Considering that with this (first) visit to Italy and the subsequent bird/mosquito experiments in Berlin, Koch had laid a good basis for a big expedition to the Far East. He proposed the expedition to the Minister of Religion, Education and Health on 29 June, 1898, with visits to several countries in East Africa, Netherlands Indies, German New Guinea, British India and Ceylon and an estimated duration of 1½-2 years. The budget request mounted to 120.000 Mark [24].

*20 October 1898 [25]: I have got the accord of my government to embark on the main expedition and prefer to spend the next malaria season in Italy again. I hope you can help me also this time, preferably in a special patient ward, where we can work without being disturbed.*

*26 November 1898 [26]: I look forward to working with you again next spring. We may already start: please have mosquitoes collected, every other week in Maccarese, and also in the hospital, and send them to me on alcohol. They have probably disappeared from living rooms and sick rooms by now and will have to be searched for in cellars, under dry leaves and grass.*

A young admirer, Giovanni Galli, supplied Koch with mosquitoes, and solicited to join him on his next expedition; Koch wrote him:

*4 February, 1899 [27]: In regard to your intention to accompany me on my imminent malaria expedition, your company and assistance would be very welcome, but I cannot offer you a paid assistant post, as those have been filled. The only option would be for you to join me at your own expense. I plan to go to Rome in early or mid-April and stay there until July or August, and then head south from there, and depending on how much the plague in Madagascar, Zanzibar disrupts communication, I will go to British India or to the African coast. The entire expedition will last about two years.*

Meanwhile, Grassi and his team repeated the Proteosoma work of Ross and Koch in humans and Anopheline mosquitoes, the results of which were published on 28 November and 22 December, 1898. Koch received the publication of Grassi around Christmas (“as a Christmas present”). His own description of the Italian expedition appeared on 2 February, 1899 in the *Deutsche med. Wochenschift* (DmW). These travel accounts were not considered a scientific publication [28], and because the reproduction of his photogrammes took a long time, the official publication, describing the morphology and transmission of Proteosoma and *Plasmodium* (*falciparum*) appeared after those from Italy [29]. Grassi wrote: “Because Koch had not yet published, we could declare that victory was ours!” Koch:

*27 February, 1899 [30]: I recently got forwarded some spiteful articles from Roman newspapers and I figure from the last publication of Grassi [31], that the jealousy and envy of the Roman malaria researchers against me have reached a rather high degree. For this reason, I do not want to get in touch with that gentleman under any circumstance. I never want to enter the S. Spirito hospital again. How about going to Grosseto or whatever other place where neither I see or hear anything from Grassi and his fellows, nor they from me?*

*19 April 1899 [32]: I would particularly like to orient myself about the conditions that precede the actual beginning of the malaria season and Grosseto seems to me particularly apt.*

## Once Again to Italy

On 13 March, 1899 the German Reichstag allowed 60,000 Mark for the main malaria expedition and on 25 April, 1899 Robert Koch with his wife arrived again in Italy. With his new crew, Prof. Paul Frosch (1860-1928) and Heinrich Ollwig, the “Stabartzt” who had joined Koch back from Dar es Salaam, he began the research in the Maremmas of Toscane near the city of Grosseto (some 160 km NW of Rome). Malaria was there at its worst from JulyOctober. Koch:

*The weather was still cool and in the Grosseto hospital we found only few cases, all relapses from last year. But on June 23 was the critical moment: fresh cases started pouring in until the malaria wards were full. This sudden start and rapid increase of the endemic was somewhat exciting. Many patients had severe malaria and we had the pleasure of not losing a single of the 330 patients. Early and correct treatment makes this disease less dangerous. The work was really fatiguing and the excursions to malaria marshes were very interesting, but we were accompanied by a carabinieri, because of the bandits in the area.*

*5 June, 1899 [33]: The only measure used by the inhabitants of Grosseto is to flee. As soon as malaria makes its vehement appearance, everybody who can afford moves. Only one tenth stays and yet, during fever time the hospital is more than full. This moment will start in the coming weeks and I am very expectantly how the circumstances will present. Here in Grosseto, there are no* Anopheles*, but in the houses in Maccarese and especially in the bedrooms it teems with* Anopheles*, overloaded with blood. Yet, none of them has parasites or sickle germs in the glands. Riddle upon riddle.*

On 10 August, the day of the patron saint Lorenzo of Grosseto, Koch received a charter from the city council. It was a gesture of recognition for his deep involvement in the experiments in the Maremma; maybe not quite suited to the fame of the person, but at any rate an important witness from the people of the Maremma [34]. Koch:

*15 August, 1899 [35]: I am very content with the work. We were able to see and mostly also treat about 500 malaria cases: I gained very useful experience for the next part of the expedition. It was very interesting to see that the fresh cases of tertian and tropical fever occur in great numbers as from a distinct point in time. The fresh tropical fever (Italian doctors call it aestivo-autumnalis fever) always are of the 48-hours type as in East Africa. It is thus real tropical fever.*

*18 August, 1899 [36]: In Grosseto I have almost guzzled in malaria and I have prepared very well for the next part of the expedition. I was able to follow almost every case from the very beginning, as has never been possible before. Malaria is to be seen as a real infectious disease, also in relation to treatment and control. In fact, it is not my intention to fight mosquitoes, but rather to try and exterminate the infectious agent (“den Infectionsstof”) in man itself.*

Koch posted his report in Naples to the DmW and sailed off to Batavia on 23 August, 1899. Koch:

*Gosio kept me informed about the course of the endemic in Grosseto: after a long peak in July-August, the number of fresh cases had diminished strikingly and ended in November. The parasites cannot stay in mosquitoes that continue to bite, as after November no fresh cases are seen. But the continued presence of recrudescing patients indicates that they carry the parasites through the inhospitable period. Thus, it is logic to treat all recrudescing patients to empty the reservoir before the suitable temperatures have returned. It is also intriguing that Grassi had come to Grosseto after my departure, obviously to repeat everything and to trumpet it out as his own research.*

Thus, Koch understood that recrudescing cases could carry the parasite through the winter, but what caused the sudden peak of primary cases in the summer? It could not be the staggering numbers of mosquitoes, because he observed none with sporozoites in the glands. It escaped him, and there were other events that required a firm stand. He had to be careful not to spread his discoveries too generously.

Indeed, Grassi visited Grosseto after Koch had left, but for other activities than Koch presumed; he stimulated the forming of a Committee for malaria control. At the end of 1900, a law was passed to start quinine prophylaxis in the area and Gosio led the campaign. Despite that, in August 1901, a new wave of malaria occurred. Anyway, it must have been a tense situation with these two Italian malariologists together; Gosio did not mention Grassi’s name in his article on the Grosseto malaria of 1899. And Grassi in his book criticised Gosio’s publication profoundly. Gosio remained active in malaria control and wrote a monograph on the topic in 1925.

After his return from the major expedition to Java and German New Guinea, Koch reported his experiences, including those in Italy that had been published already on September 14^th^, at a meeting of the Deutsche Kolonial-Gesellschaft (BerlinCharlottenburg) [37].

Meanwhile, Ronald Ross was kept updated about the activities abroad. His initial contender was Koch, whilst Grassi´s publications were brought to his attention only during the next year. Ross received a letter from Rome (4 November 1898) [38]:

*Koch is not in Rome now, but though I tried hard to find out what he had been doing I can get hardly any information. He seems to have been very silent as to his work and to have refused to speak about it. It may interest you, however, to know that he spoke in the highest terms regarding your work and also that he looks upon the flagellum as being some kind of spore.*

Another informant, the parasitologist George Nuttall, who reviewed in Berlin the progress of malaria work in German medical journals, and knew that Koch had already proposed the mosquito-theory while on his cholera expedition in 1883-1884, had brought some specimens of Grassi to Berlin in March 1899, but Koch did not bother to examine them [39]. A bit of flexibility at that moment would have changed malaria history… From then on Nuttall turned his back to Koch, and wrote on 13 August, 1898 to Ross in Assam, India [40]:

*Koch, who came back recently from German East Africa, declares himself in favour of the [mosquito-malaria] theory and says it would be very important to follow up the matter experimentally on mosquitoes. He ignores your work completely.*

And again on 21 December:

*I heard the other day that the Koch institute is full of birds and mosquitoes. They are awfully suspicious and secretive.*

Ross replied:

*I did not like to hear all this of Koch, who was to me a star of the first magnitude, and I could not conceive how he or any man of fame could plagiarise the work of an obscure person like myself. But it was evident that scientific morality was not as impeccable as I thought.*

Ross to Manson, Calcutta, 2 February, 1899:

*Please don’t believe too much of Grassi & Cos’ work. There are passages that make me think that their actual observations have been of the slenderest and that the rest is eked out by aid of my reports.*

The scientific competition had begun to become grim. Meanwhile Koch was sailing to Batavia, the capital of the Netherlands Indies.

Koch observed the ecological behaviour of malaria transmission: before the end of June only relapses of previous infections; thereafter a sudden wave of primary cases of aestivo-autumnal fevers (= tropical malaria). He followed many patients individually, much more meticulously than is the practice nowadays; we hardly recognise a tertiary fever rhythm in patients infected with *Plasmodium falciparum*. The relation with mosquito-infections in human dwellings is still enigmatic for him.

## Part III: The Expedition to Java and New Guinea

At the age of 56 years "Geheimer Medizinal-Rath" [a high-ranking medical position], Koch was placed at the head of a malaria-expedition, fit out by the German Imperial Reich to visit Italy, Java and German New Guinea. Koch was a corresponding member of the Society for the Advancement of Medical Sciences in the Netherlands Indies since 1884 and he had heard and read that malaria was especially severe in Batavia (Jakarta today). I have traced Koch’s activities in the medical journal of the Netherlands-Indies as well as in the daily newspapers of Java. The compilation of Koch’s reports is presented through free translation of selected passages, to allow the readers to appreciate his reasoning, decisions and statements as closely as possible. I also followed the events occurring in the slipstream of his visit to Java [41].

Koch, his wife Helga and his assistant, military doctor Ollwig arrived in Batavia on 21 September, 1899 and they stayed until 12 December. Some days later the board of the Society paid their respect by visiting Koch in his hotel ‘De Nederlanden’. The bacteriologist J. de Haan reported: “In accordance with the Governments desire, all wanted help was offered and a part of the Laboratory for Pathology and Bacteriology in Weltevreden [southern suburb of Batavia] put at their disposal”. Subsequently, the medical officers in charge of civil hospitals and military garrisons were ordered to supply the Professor with all help, including catches of mosquitoes in those buildings. Even the patients contributed, by allowing biting mosquitoes to feed undisturbed.

On recommendation of Koch glass tubes were used for catching. The professor was informed about suspected malaria patients in the nearby hospitals and mosquitoes were brought along. Data from the Indian literature and statistics were collected and presented.

The Batavian Newsletter reported on 29 September:

*We are told that Professor Koch has said that nowhere abroad he had been honoured with such a warm and generous reception as here in our colonies. Especially the Memoir concerning the experience with malaria in this country, written in German and offered to him right after his arrival, has been a very pleasant surprise. He felt overwhelmed by all the efforts to inform him in details.*

Just in that week (28 September) the influential Java-Bode published a detailed review from an Italian newspaper on the progress that Professor Grassi had made with his malaria studies and the experiments proving that *Anopheles* mosquitoes were the vectors of human malaria parasites. No introductory statement was given in relation to the arrival of Professor Koch and his malaria studies in Java. It must have been brought to Koch’s attention, but he did not refer to it in any of his letters or other writings. We may only guess about his feelings, to be haunted with Grassi, at the other end of the world...

Koch sent his report about the Java experience to the DmW [42]:

*Searches for parasites were carried out among Batavian doctors and in the hospitals; we got only 30 patients during five weeks. Also, in the colonial army there appeared a major decrease in malaria patients. Apparently, it plays by far not the role as before, and as I had presumed from readings. People consider the improvement of drinking water as the cause, but this is incorrect, because the harbour of Batavia, renowned for its malaria, has still a lot of malaria, as well as tap water from artesian sources.*

*Cost-free distribution of quinine has in my firm opinion contributed to the lowering of malaria. 2000 kg is yearly made available to the army and the civil population, including the locals. Countless malaria germs must have been destroyed in this way. The free distribution is a very recommendable measure, and all malaria countries are strongly advised to follow the example of the Netherlands Indies [43].*

*The time in Batavia was used to solve the important question whether malaria was transferable to other animals. I have thus bought or borrowed apes and monkeys (3 Orangs and 4 Hylobates), and injected them with infected human blood. None of the animals did come down with tertian or tropical fever, or with parasites in their blood. Man remains thus the only carrier of this parasite, a fact of greatest importance for the prophylaxis [44].*

The Java-Bode wrote on 6 October:

*“Prof. Koch seems to be a great animal friend. He is at present the happy owner of an orang-utan that has been chummed up with him, thanks to his loving care. Yet it will have to serve as a guinea pig for malaria research.”*

On 22 October, 1899 Koch wrote to Gosio in Rome [45]:

*We have been able to do few malaria studies: in Batavia only six fresh cases, mostly tertian. The situation in Batavia has markedly improved over the last 10-15 years. There is a lot of* Anopheles *here (of another species than in Grosseto), but I failed to detect sickle germs, even in those fed on blood with crescents. Soon I will get inland to visit sanatoria and hospitals.*

As a whole, the medical society there did not excel in knowledge on malaria, but Koch, “the king of science” was appreciated for his "high point of view in our science", his eloquence and clearness. Koch in turn was courteous towards the board of the Society, when invited to deliver a lecture. That happened on 26 October, before a plenary gathering of members. He lectured about the aetiology of malaria and the state of knowledge of the malaria problem, including the transmission results of Ross with bird malaria. He showed a series of highly interesting shadow pictures of microphotographs from his own bird-malaria studies on the wall with the new projection apparatus of the Society [46].

The Java-Bode reported the details of this lecture (28 October):

*The theory of miasmic intoxication was disproved by sub-inoculation experiments and Laveran described the parasites. Golgi [47] demonstrated that there are several parasite species, each with its distinct clinical course. The tropical fever and the summer-autumn fevers appeared to be caused by the same species, as Koch had demonstrated. In Batavia he found about equal numbers of tertian and tropical malaria, which resembled more the Italian and less the African situation. With respect to the infection, Koch denied the possibility of parasites in dust or water and referred to the experiments by Ross and himself on bird malaria and Grassi [!] of transmission by (anopheline) mosquitoes that have led to the mosquito theory. The speaker rejected common counter arguments of areas with mosquitoes but without malaria. He launched a much more important question: are there areas with malaria but without mosquitoes? He had seen such conditions in East-African mountainous areas. Malaria cases in the mountains occurred invariably within 10 days after travel through malarious lowlands. The speaker invited his audience to supply him with information about such areas in Java.*

**Figure 5.**
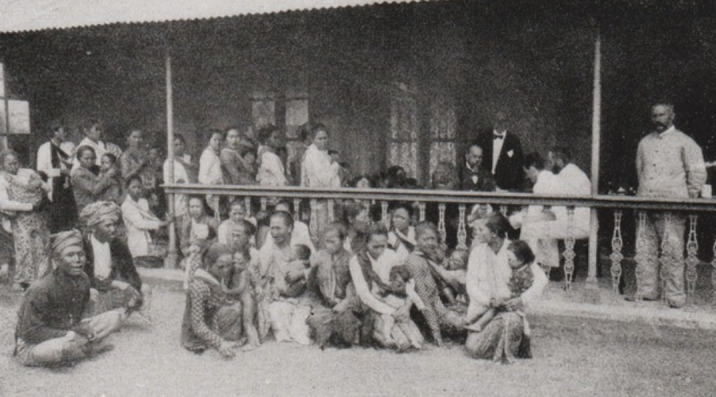
Ambarawa hospital: mothers with their young children; Koch sitting behind the fence.

The number of malaria patients being disappointingly few in and around Batavia, a field trip was planned. The head of Military and Civil Service ordered the lecturer at the Dokter Djawa Medical School, military doctor J.J. Kunst, to assist Koch in his research and to join him during the travels through Java, on governments’ expense. The expedition left Batavia on 28 October for Ambarawa in central Java, south of Semarang, because there was a military hospital with research facilities. Via Bandung, the extensive Cinchona gardens in Lembang and the modern Tjimahi hospital the party went eastward. A day was spent at the Borobudur. And via a visit to the hospital of Magelang they arrived in Ambarawa (Figure 5). Koch:

*In spite of careful searching during two weeks we could find only 21 real cases, but the environment was so suitable for malaria, that I decided to examine children, pounding the possibility of a certain degree of immunity among adults, as I had seen in East Africa. We chose a village in a marshy area, where the adults considered fever of no burden. 9% of the children were found infected and among the infants 16%; in another village the figures were even 23 and 41%! I judge these results of great importance. We have now a method with which one can obtain absolutely dependable information about the malaria situation at short notice. Malaria appears to be a children’s disease and the young age classes are perfect indicators for endemicity.*

A newspaper journalist heard Koch sighing: “*There must be one point on Java, where no malaria occurs*”. And Koch wrote:

*The next goal was to do similar studies in a malaria free area, and as such I was advised to go to Tosari, at an elevation of 1777 m in the Tengger Mountains of East Java. I was told that there was some malaria, but no mosquitoes. On 26th November we reached Tosari. In none of 82 children we found malaria parasites, but an adult patient in the hospital did have parasites. Twelve days before getting sick, this man had gone down to the coastal valley where malaria is endemic, where he spent one night. As we found no mosquitoes in Tosari, this situation was very similar to what I have seen in the Usambara Mountains in East Africa. The sporadic cases are imported ("eingeschleppt"). I have enquired among many older and experienced doctors about places without mosquitoes, but nobody could indicate a mosquito-free area in Java. Close examination always yielded mosquitoes. The Tengger Mountains are the only exception. It is another proof of the new mosquito theory, and one can safely state “no endemic malaria without mosquitoes”.*

In the malaria sanatorium of Tosari, Koch disputed the usefulness of sending malaria sufferers to sanatoria in the mountains and he commented:

*Once infected, the climatic conditions at certain elevations cannot prevent the attack or relapse of malaria among reconvalescent patients. And in none of the sanatoria the recovery of malaria is exclusively left to climatic healing, but strong quinine treatments are applied everywhere. So, it is my conviction that the mountain climate has no specific action upon the malaria parasites, whatsoever.*

The newspaper Lokomotief quoted the savant on December 12, talking about the use of quinine:

*”Everyone with a gun can shoot, but not everyone can hit” and the journalist concluded that “Many doctors in the Indies are absolutely not convinced that Koch’s mosquito theory is THE solution.”*

Dr. Kunst wrote later [48]:

*Great was the commotion in the camp of the orthodox doctors, when Koch stated that transfer of malaria patients to a cool and malaria-free climate would not have the least influence on their cure.*

Instead, reconvalescents could experience relapses at high elevation, despite intensive quinine treatment. This was considered a paradoxical, unbelievable statement. Many discussions with the colleagues, also the in other sanatoria visited, ended in a tie, because Koch demanded microscopical proof, which they could not give. Also, in several other localities virtually no malaria was found, much less than in the opinion of local doctors. Koch:

*In Soekaboemi we were offered 780 children of whom we selected 200. One was not sure what to admire more: the docility of the population or the influence of the authorities and the efficient organisation.*

*Upon our return to Batavia, Colonel De Freijtag offered huge numbers of anopheline mosquitoes, coined by the Italian researchers in 1898 as the transmitting genus. Now I can already convey that the mosquito fauna of the Netherlands Indies is indeed manifold. I have got at least five* Anopheles *species, mostly associated with rice cultivation, though I was not successful in catching larvae in the paddies. Unfortunately, we have also not succeeded to discover the known Coccidia on the stomachs or the sickle germs in the poison gland of many* Anopheles *and other mosquitoes from malaria-ridden Tandjong-Priok (Batavia harbour). These forms even failed in* Anopheles *after feeding blood with malaria parasites or containing the crescent parasites. In Tandjong-Priok 38% of the infants had malaria parasites!*

*Remarkable is that we have not found other types of malaria in the Netherlands Indies than the three known ones, however, the dangerous “tropical fever” occurs here less than in tropical Africa, where it reaches 87%. Of all malaria cases in Java, 47% were of the tropical fever type, 45% was tertian malaria and 8% quartan malaria.*

Koch was very satisfied with the help of his assistant Kunst and praised him for his indefatigable diligence and helpfulness. Thanks to this Koch finished his Java-report in Batavia on 9 December. The board of the Society considered it discourteous to invite Koch again to present the expeditions’ results "in view of his many worries during the few days left before sailing-off to New Guinea". The Batavian medical circle did not have to wait too long for the news: it appeared on 1 February 1900 in the DmW.

The expedition left Java for German New Guinea on 12 December. Looking back, Koch considered [49]:

*We got the opportunity to get to know and compare subsequently two very interesting colonies in the tropics in some detail. Java was an old colonial possession, for several centuries under European influence: a heavily populated land with railways and roads throughout, with rice cultivation and other plantations replacing the forests. New Guinea on the other hand, was a fresh, almost untouched land, sparsely populated and covered with forest.*

## Early Reactions

Dr. Kunst had become a devoted disciple and professional admirer of Koch. After the departure of his teacher, Kunst continued pleading for microscopical examination of blood. Koch had left him almost 1000 slides! But most medical doctors in Java considered this as busying with scientific trifles ("Spielerei") and unsuitable for the daily practice. He took the lead in the Military Hospital of Weltevreden and found many fever cases without parasites (only 177/600 were microscopically positive; many non-malaria cases turned out to have typhoid fever!). Kunst was convinced that, thanks to Koch’s visit, no doctor in the course of time would consider treating without microscopical examination... The extensive (84 pages) and knowledgeable paper gave an interesting and sympathetic commentary of Koch’s scientific activities [50].

Other medical doctors were more critical, and the military medical officer Van der Burg, referring to the few children found infected, declared it an error of Koch, because he had arranged to call up the children to appear before the physician. The people regarded such a summon 'en masse' as a vaccination round and only the healthy children would have appeared!

Following the wave of more or less grumbling reactions, initiatives were taken to bring the statements of Koch to the test, which gradually changed the attitude towards malaria into a more scientific way.

The find of many infected children, in contrast to adults, led Koch to conclude that they are the key to endemicity and immunity. At high elevation he found neither mosquitoes nor patients, which matched the East African situation. He disputed that patients improved in clean air; only quinine helped.

## German New Guinea

Koch settled in Stephansort (now Madang, Papua New Guinea) and went for the aim of freeing the town from malaria. He stayed for half a year and sent his reports to the DmW, where they appeared as a feuilleton during most of the 26^th^ volume in 1900. I have picked citations that dealt with malaria proper:

*Malaria is the heaviest burden that hampers the development of our colony. It lies like a poisonous vapour on this beautiful lustrous land. Virtually all Europeans get sick within some weeks; they die or have to repatriate within two years. The same holds for the foreign workers from China and Malaysia [51].*

*January-February, 1900 [52]: We chose Stephansort for our longer stay, because it had tobacco-plantations, about 600 workers and two hospitals, one for Europeans and one for coloured people. The soil is well drained and there are no marshes.* Anopheles *mosquitoes and malaria are abundant, particularly in the rainy season, at the beginning of which we arrived.*

*Within two months we saw 21.4% of 734 sick people with malaria parasites in their blood. Probably this is an underestimation as parasites may temporarily cease to be present in finger blood.*

*All three types of malaria were present and more frequently amongst imported workers than in local inhabitants. We treated all parasitized people and after two months in the rainy season we found only rare cases during our control examinations.*

*Another fact that can be concluded to my knowledge here for the first time in a very convincing way, is the natural immunity that people from tropical malarious areas acquire in a few years. The villages Bogadjim and Bongu along the Astrolabe Bay give classical proof. Children between 5-10 years of age show little to no malaria. If one had consented oneself with examining the adults, one would presumably not find a trace of malaria and lead to the utterly wrong idea of a population without malaria. This phenomenon needs to be confirmed among indigenous people, both in the Heimat and in the German Colonies, especially in East and West-Africa.*

*3 May, 1900: Immigrants from non-malarious areas would behave like children in endemic areas. The patterns of Europeans, Chinese and Melanesian workers are very instructive. Europeans generally get their first fever attack 3-4 weeks after arrival. The exceptions are the two members of the expedition, undoubtedly because of their consequent quinine prophylaxis [53].*

*Among the Chinese workers, who lived here since nine to four years we found only 4,6% sick, but of the coolies who had arrived more recently, 42% had malaria and from those arriving at the same time as the expedition 70% suffered. Within one year, 46% of the imported Hong-Kong Chinese died, mostly of malaria. So, our investigations about the present malaria situation in N-G went hand in hand with our efforts to master the malaria as much as possible, i.e. to exterminate the parasites in man.*

*Not only we treated patients, but we also tried to prevent recrudescences (“Recidive”). I had already some experience with people in Berlin, after having returned from the tropics: a dose of at least 1 gram, for two days.*

*Our findings here were that a break of seven days between two quinine days is completely sufficient to prevent malaria setbacks if maintained for at least two months. Treatment according to this principle has given a considerable reduction of malaria among the coloured workers, despite the climatic conditions that are so favourable for malaria and* Anopheles *mosquitoes.*

**Figure 6.**
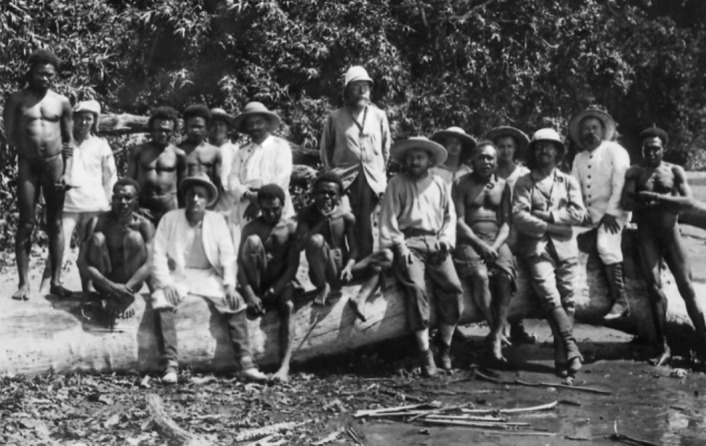
The crew, amongst Papuans.


*Stephansort, 28 April, 1900 [54]: By the measures that we took, the malaria has thus been reduced to a minimum, during the rainy period that used to be most unfavourable. We are presently in the highly feared period.*

*A group of people from the island of Ambon came to Friedrich-Wilhelmshafen. It is known that Ambonians are very sensitive to malaria. Half of them got prophylactic quinine, the other half not; the first group remained healthy, whilst almost all in the other group got ill of malaria. The latter were brought to Stephansort and recovered after treatment.*

*Both members of the expedition are still, after four months, exempt of malaria, due to a regular quinine prophylaxis. One sees from this that susceptible people in a malaria-ridden environment can be protected with certainty, despite the fact that the use of quinine is somewhat cumbersome and for many people even quite unpleasant.*

*I might call attention to one important condition that appeared in our investigations. There are people that hardly have clinical symptoms but in whose blood parasites can be demonstrated. These chronic cases may have gone through a series of relapses and do not suffer from fever anymore. They have no reason to visit a doctor. This means that only a fraction of malaria parasites in a population will be terminated. There is no other way to identify all hidden cases amongst suspected people (children and newcomers) through regular blood-examination and treat the positive ones. A tiresome and time-consuming procedure, but I would not know how to proceed otherwise, in order to suppress malaria rapidly and surely. With all these experiences I feel justified to state that it is possible to make an environment free of malaria, provided enough doctors and quinine. It will be less easy in uncivilised areas, where the inhabitants reject European medicine. The locals are not easy to convince to take drugs, but thanks to talking and small presents I succeeded several times to get people to give quinine to their children.*

In April, Koch took the opportunity to make a boat trip to the western coast of the colony and the islands. He visited Finchhaven, the coast of the Gulf of Huan, the Siassi Islands, Mantok, Aramut, the coast of New Pommern and the French Islands (Merite and Deslacs). In some places he found virtually all children with malaria and in others none. He concluded that it is of paramount importance for the recruitment of coolies, to know the dispersion of malaria.

*Stephansort, 15 June, 1900 [55]: During six weeks we had only four cases of malaria, all quartan, the most stubborn type. Almost six months of experimental control has brought malaria almost to disappearance.*

*It may be that other methods are also suitable for control. One may consider to speed up the development of immunity, which takes 4-6 years and many attacks. But so far the required poison cannot be cultivated and thus, this is not a method for the near future. Also, the extermination of infected mosquitoes in larger areas is in my view beyond the power of mankind.*

*Finally, one could try to protect against mosquito bites, like the use of widely known mosquito nets. Provided it has no holes and can be closed well, and people stay inside from dusk to dawn, it gives full protection. In Stephansort every worker has his net and they know very well how to use it. Yet, it has had no influence on malaria, simply because the workers don’t go to bed at dusk but go after their pleasures until deep in the night.*

*Also oils that repulse mosquitoes work only for a few hours in my experience and may be harmful when used for long time.*

*The only method that works is the one I proposed, namely to trace all cases, and particularly the hidden ones, and to treat them thoroughly. The use of quinine for long periods is hard to support, but for those who are on expedition, who pass a malarious harbour or stay for a short time in a malarious area, quinine prophylaxis is to be recommended strongly. In terms of control, it is only a minor, restricted method, compared to my approach of treating all parasite carriers, diseased and healthy alike.*

Koch left Stephansort for Herbertshöhe (Kokopo, New Britain), the seat of the Imperial Government of German New Guinea; there, he recovered from the hard work.

He left on 6 August and the boat trip to Germany gave Koch the opportunity to visit the Caroline and Marianas Islands in the North Pacific [56]. On the islands of Ponape and Saipan he found none of some 100 children with malaria parasites or enlarged spleen and concluded that the island was malaria-free. Back in Berlin Koch wrote to Pfeiffer on 10 November, 1900:

*We had to work very hard to perform the necessary mass-examinations. On top of that I had to replace the plantation doctor, who was sick and was repatriated, but died underway. Also during my trips through the South Sea of two months I had to act as ships doctor. The expedition was not a holiday trip, but looking back it was highly interesting [57].*

The editor of the DmW asked Koch to summarise his somewhat fragmented accounts [58] I have selected some items that he highlighted about New Guinea:

*Among the islands of the Bismarck Archipelago I found some exclusively with quartan malaria. Workers brought from there to Stephansort got tropical and tertian malaria. Thus, one type of malaria does not protect against the others.*

*In order to master malaria, we must send many more doctors, who are trained in microscopy and in completely curing malaria until no relapses occur. They must make quinine freely available to the poor, like they do in the Netherlands-Indies. Only then, we will gradually reach our goal, and eradicate the malaria, which hampers the rapid development of our colonies.*

*I must emphatically state that this treatment should not be confused with the long-known quinine prophylaxis, which only prevents infection in the individual and is of no value for malaria control. My measures are directed against the sick and all those can be treated, thereby rooting out the malaria.*

Koch ended his overview with technical aspects of treatment and prophylaxis. He recognised that the agents of Italian and tropical malaria were the same.

The achievements of Koch appeared not sustainable, due to lack of funds and organisation amongst the German Colonial Association and the missionaries; unlike the situation in Java, there was no scientific follow-up in German New-Guinea.

Ewers remarked that the works of Robert Koch on malaria has had insufficient recognition in the English-speaking world. He went at length in recounting Koch’s activities in New Guinea and his contribution to malariology [59]. Later, the medical historian Eckart explained in his essays about Koch and the control of malaria in German New-Guinea, why this colony was the end-goal of the malaria expedition [60]. It was the interest of trade, resources, German officials and missionaries. A review of Koch’s discoveries reveals the topicality in modern research in Papua New Guinea [61].

In highly endemic New Guinea, imported workers suffered, like children; one type of malaria does not protect against others. Two-day cure every week, after recovery with quinine, prevents relapses (proved with control group!). Individual prophylaxis does not contribute to control. Intact bednets are of use, provided they are used when mosquitoes are active.

## Part IV: Malaria Control in Istria

Already during his stay in New Guinea Koch thought it useful to have a malaria study site in Germany, to repeat his studies and he asked Prof. Frosch, who had accompanied him on his second visit to Italy, to travel around. It appeared that malaria was rapidly retreating and even in the marshy areas along the German North Sea coast he found only dispersed cases. Nowhere a suitable malaria herd was found.

Shortly after Koch returned, Paul Kupelwieser, the Austrian owner and governor of the island of Brioni approached him. He had read in the newspapers about Koch’s activities in Grosseto and requested his help to control the malaria on this small island with 300 inhabitants (Brijuni, before the Adriatic coast of Istria peninsula, Croatia). He wanted to transform it into an archaeological park and spa resort, but malaria was in the way.

Koch saw his chance, reacted quickly and one week after the request he sent Frosch and an assistant, Dr. Elsner, to the island. Frosch immediately started to take blood smears from finger-pricks and found among 253 smears 20 cases of tropica, 20 cases of tertian and 1 case of quartan malaria. One week later Koch himself set foot on the island. He planned experiments to control malaria through quinine, as he had done in New Guinea.

Kupelwieser, not a medical man, gave himself major efforts to learn from the world-famous Koch and he started to make slides, which he sent to Berlin. But his superiors in Vienna were less pleased, and considered it an inappropriate initiative to invite Koch: Austrian doctors, in particular the malaria expert Dr. Mannaberg could have done the job…[62].

Koch sent more doctors to take a course on malaria, given by Frosch, to prepare for their work in the colonies. Koch visited the island again and also the neighbouring mainland of Istria on 5 March, 1901. Wisely, he first passed through Vienna, to convince the section head of the Ministry about the studies at the island and got full support of the Austrian authorities [63]. Koch:

*20 March, 1901 [64]: Thank you for the blood smears that arrived here in good condition. With our 5 or 6 microscopists we have finished 161 smears, with only ten positives. Progress is a little slow, as I had told the gentlemen that careful work is more important than quick work. Don’t forget to arrange everything for the quinine treatments, and make sure there is always somebody to do the follow-up of the patient and the blood smears.*

*Berlin 24 April, 1901: The malaria cases so far confirmed and treated with quinine is 63/383 or 16.4%. Most of them continued using quinine for three months and therefore it became important for me to convince myself about the result of the course. Immediately after arrival we also arranged to study the malaria-ridden coastal villages. Stignano 8.3% malaria diseased people, Fasana 6.8% and Peroi only 1.4%. I considered it important also to start treatment in the two first villages, to avoid reintroduction of the infectious material into Brioni through traffic and possibly also through airstreams.*

*Whilst in Pola [town on opposite mainland], I was urgently invited also to study the villages of Ossero and Punta Croce near Lussinpiccolo, which were supposed to be highly malarious [villages on islands east of Istria]. We found 4.7% and 10.7% of the population to be infected. Because of the isolated situation of Punta Croce, I considered it necessary to station military doctor Bludau in Lussinpiccolo. I will pay the costs out of the means of the Malaria expedition. I am pleased to mention that the Austrian authorities have taken the distribution of quinine to Punta Croce and other villages upon themselves.*

*On the way back I visited Rovigno with the zoological station of the Berlin Aquarium, to see if malaria studies could also be organised from there. I found out that attempts had already started here to destroy mosquitoes, following the Italian ideas. As it is not useful to try two procedures based on different principles, I have resigned to undertake something in Rovigno.*

*4 June and 19 July, 1901 [65]: Our studies in Istria are still in full swing. The Brioni islands are now free of malaria and in 4-6 weeks we will see whether our experiment to exterminate malaria has succeeded, because then the malaria period starts and the fresh cases appear in bigger numbers. We are close to the unravelling.*

On 25 June, 1901 Koch delivered the Harben Lecture in Eastbourne (UK), on the various methods of malaria control and his project in Istria [66]. After having considered all measures aiming at exterminating the parasites as yet to be proven in the field, his own approach with quinine treatment and prophylaxis had stood the test already. Before elaborating on the Istria experience, Koch emphasised the importance of getting rid of the parasites in every person attacked by malaria, as well as those with chronic malaria, in whose blood parasites can hardly be demonstrated and who have nearly no perceptible symptoms:

*To render all -or as nearly as possible all- parasites innocuous, we must examine the blood of all suspected persons with the microscope, before treating with quinine. In all the attempts I have hitherto made to exterminate the malaria-parasites, I have acted on this principle, and have been able to convince myself that the execution of this measure is not so difficult as it may at first sight appear… The taking of the blood for examination is so simple and purely mechanical a matter that no medical doctor is needed for it. The blood-preparations made by sick-nurses of both sexes and other non-medical persons were very satisfactory… So it is not at all necessary for a doctor to do all the work himself. With a sufficient staff of such assistants, a single doctor will be able to rule a pretty large malarial district, and rid it of the parasites… The well-known optical firms of Zeiβ and Leitz have recently produced small microscopes, perfectly sufficient for this purpose, costing ₤ 15 to ₤ 20…*

*I regard such thorough investigations of the population in malarial districts as absolutely necessary.*

*Istria: The malaria corresponds exactly to the traffic that goes on in the various places. In Punta Croce and Ossero, at the southern end of the island of Cherso and at a distance of all traffic, only the children have malaria- parasites in their blood, exactly as I found it in New Guinea and Java.*

*Stignano and Fassana near the town of Pola are not so entirely cut off from all traffic and the population is more fluctuating. There, malaria is more frequent among the older people.*

*At Brioni, the population consists almost entirely of workmen who come to the place and leave it again in swarms. They come from the most different parts of Dalmatia and from the mountainous districts of Istria, which are free of malaria. Almost all get malaria at Brioni, the consequence of which is that the majority of the malaria patients there are adults. In Fassana and Stignano the malaria cases were especially numerous in certain houses and groups of houses. From this we may conclude that the infecting mosquitoes do not fly anywhere and everywhere, but have predilections for certain places. From this focal behaviour of malaria, I drew the practical conclusion that it is not necessary at the outset to free whole areas, but we can advance step by step, without having to fear that the ground just freed of malaria will be at once re-inundated by infected mosquitoes.*

*Based on the very many experiments I have made, I order 1 gramme or 15 grains of quinine to be given two mornings running, which is repeated after an interval of nine days. This treatment must be continued for at least two months. In quartan fever, which is well known to be the most obstinate form of malaria, quinine must be given three days running from the first. To children under six months one generally gives 1/10th of a gramme, i.e. 1½ grains, to older ones according to their age. They generally stand quinine very well. They do not dislike it either, if given as a powder mixed with raspberry syrup, or if sweet tea or the like is given after it.*

Koch received the Harben medal for his outstanding service to public health.

*12 December, 1901 [67]: This year there were only 17 fresh cases on Brioni, against some 100 in previous years. As in nearby Pola the malaria had its regular course, the moderate appearance in Brioni can only be attributed to our control measures.*

*Whether the fresh cases in July and August have arisen from* Anopheles *which had taken up the malaria germs in the previous year, or from the few cases in spring of this year, is not certain.*

*26 March, 1902 [68]: We found in Istria that children can support much larger doses of quinine than we have assumed before, and they need it. I hope we can round off the work in Brioni next summer and publish the results.*

*18 October, 1902 [69]: The complete success has pleased me greatly, because it proofs that the original ideas were right and that malaria can be exterminated by this method. Now I think it is feasible to free larger areas from malaria within some years and without too many costs, for example South Istria and the city of Pola. But first and foremost, you have to take care to sustain the situation and I am fully prepared to support you in this.*

*18 November, 1902: Similar to what I did in Stephansort, namely identifying malaria parasites among all people and treat positive cases, trials in Istria on the Brioni islands and in the villages of Punta Croce and Ossero also gave good results. The malaria has been completely wiped out in Brioni with not a single fresh case since 5 trimesters, whilst in the neighbouring areas, malaria goes on unaltered.*

**Figure 7.**
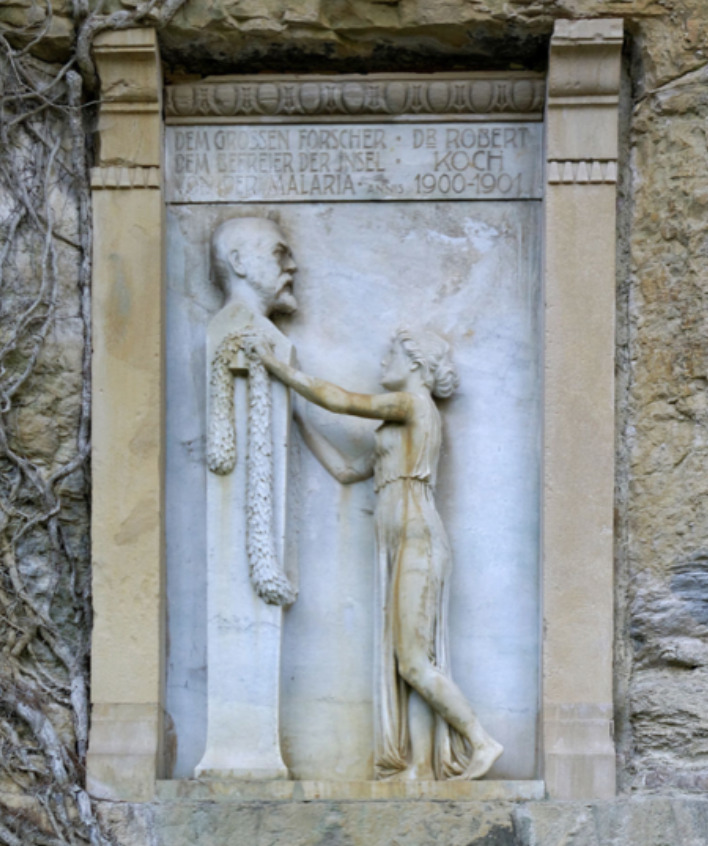
The memorial at Brioni, placed in 1909.

Kupelwieser condensed his acquired knowledge in a field/lab guide, which he had corrected by Koch. Much to his disappointment, the Austrian minister of health did not consider it proper for use by medical doctors, because Kupelwieser was merely a lay. Kupelwieser succeeded to distribute it on his own account and completely sold out. He expressed his gratitude for his teacher by erecting a relief in Brioni (made by Josef Engelhart) for the great benefactor of mankind (Figure 7) and he described in detail his memories of Koch in his autobiography (1918).

It must be admitted that the drug mediated control method of Koch was successful in the small Brioni island, and after Koch consented to fill, oil and burn the mosquito breeding pools, extirpation of malaria appeared sustainable: neither Kupelwieser, nor Dr. Otto Lenz, who started as island practitioner in 1903 (till 1938) seemed to have diagnosed any malaria transmission on Brioni, whilst the tourist industry boomed and malaria remained endemic in mainland Istria.

Normally, malaria control officers on an island must have taken into account that after a successful quinine-campaign, a less strict regimen would eventually allow for imported cases (and anopheline mosquitoes) from the mainland. Moreover, tertian malaria can survive quinine, and relapse after the interruption of treatment. Control with quinine has long been abandoned, but also modern drugs as single means are insufficient to extirpate malaria from a geographically isolated area.

## Part V: Conspiracies in a Priority Battle

The activities of Koch in Italy had caused bad blood among the other Italians who studied malaria. Giovanni Battista Grassi and his co-workers felt overshadowed by the success of Koch. Grassi complained bitterly that Koch behaved as Caesar: "Veni, vidi, vici". And then there also was this British doctor Ross, who could not even tell one mosquito species from another.

As we have seen earlier, Koch had not been particularly keen to honour those who earned it. The race was on, and pressure of time forced all of the contenders to publish as quickly as possible. The results were not always very well equilibrated.

The episode of the priority race has been reviewed many times; this time Robert Koch is presented as the genius behind the curtains. As from his Indian period, Ronald Ross was kept updated about the activities abroad. His initial contender was Koch, whilst Grassi´s publications were brought to his attention only during the next year. After his return from the main malaria expedition, the statements of Grassi again bothered Koch. Grassi had published a book in 1900, which appeared in July 1901 in German translation: *Die Malaria. Studien eines Zoologen*, with exquisite illustrations in full colour. In his Historical Notes, Grassi extensively unfolded his discoveries versus those of Ross and Koch. It must have stiffened Koch in his feelings for Grassi, and no word of appreciation for the scientific or artistic merit of this monograph was uttered. Also, Manson and Ross were disturbed about its polemical style. As a consequence, the relationship between Koch and Ross became much more collegial and Ross took Koch in protection [70] , by stating that:

*…because Grassi, when comparing with his own work, stubbornly gives false impressions about Koch’s contribution to these investigations.*

Koch complained to his British colleague in a letter of 10 February, 1901 [71]:

*…although I consider Grassi to be a rogue and a robber in scientific domains, I should not pass over his scientific merits where they ought to be mentioned. But it is my conviction that he has no such merits. What he claims as his, is either stolen or fabricated… His statements regarding the development of the malaria parasites in the body of the mosquito, if he really has seen them as he states (which, by the way, I do not believe) are only a confirmation of your discoveries. His illustrations are nothing more than copies of yours. The first infection experiments which were made in Rome by Grassi and his collaborators, and so very loudly advertised to the entire world, I consider to be inventions; for they were made in a season during which there are no fresh infections in Italy…. It seems to me that one has to be very careful and also very sceptical as regards the so-called Roman School.*

Ross consulted Dr. Laveran, the discoverer of the malaria parasite and included a copy of the above letter by Koch. Ross by then considered both Laveran and Koch as his intellectual forebears, next to Manson [72]. Laveran answered on 26 March 1901:

*Those who have followed with attention your investigations, know how to consider the assertions of Grassi…you do thus well to defend yourself and unmask the pitiful and infamous methods of those Italian authors who seek to belittle the importance of your work to their own profit.*

Koch to Ross, 15 May, 1901:

*I got convinced about the mosquito as transmitter of the malaria-infection during my visit to British India (winter 1883/84) and have ever since expressed my views in this sense… At any rate, I have not put a great value to emphasise my priority in this case, [i.e. the Proteosoma transmission experiments] because it concerned only confirmation of already known things.*

*Completely different is the situation in your battle for priority against Grassi, in which you must not under any circumstance stay behind…*

*I recently had to explain to a member of the Jury of the Nobel Prize that the important discovery of the courses of development of the parasite in the mosquito is exclusively thanks to you and ... that the Italian researchers are as little involved in it as Manson. I can only inform you about this most confidentially.*

On 20 July, 1901 Koch went to London to attend the British Congress on Tuberculosis and on the 25th he delivered the above-mentioned Harben Lecture in Eastbourne, on the various ways of malaria control. Thanks to this visit and Koch’s’ lobbying activities (while Ross had just left for Sierra Leone), Ross was solely awarded the Nobel Prize in 1902 and among the 41 other contenders Grassi was excluded [73].

Meanwhile, Koch himself had suffered from malaria [74].

*I suffer from malaria. After a period of prodromal signs I got several attacks and found tertian parasites. Of course, I took quinine, which I did not support well. I don’t know where I got it. Either from a trip to the Ruhr area or it is a relapse from earlier malaria last year.*

Ross congratulated Koch in 1905 that he too was honoured with the Nobel Prize and asked his recommendation as candidate for the position as Professor in Protozoology at the University of London. Koch did this on 18 December, 1905 in most favourable wording and with regard to the Nobel Prize he wrote:

*Through this we have become colleagues in double ways.*

In the priority race about the mosquito as vector, and as essential environment for the parasite’s development, Ross, Grassi and Koch each had their discoveries. Grassi did not grant the others their part. Koch eventually accepted to be the confirmer of the results of Ross, and joined him in the slanging-match against Grassi. The evidence that Koch manipulated a member of the Nobel committee is brought forward here, in his, so far unpublished letter to Ross of 15 May, 1901.

## Concluding Remarks

With this exposé, I wanted to share my experience on malaria and mosquitoes, both in the laboratory and in the field, and compare them with Koch’s letters and publications. Time and again, it was a shock of recognition: he understood what he saw. He discovered details that we tend to ignore and he drew conclusions, that reached far. At the same time, it may be humbling to compare our own research with his, hundred and twenty years ago.

Initially Koch has balanced on the edge of suggesting more than he could prove, with regard to the mosquito theory, but his activities certainly spurred his contenders to press on. He soon gave Ross full credit as the first to prove the mosquito theory and gradually, he contented himself with the role of confirmer of the Proteosoma experiments. Koch’s original description of worm-like structures in the midgut of mosquitoes (the ookinetes of Proteosoma) was later well acknowledged by Ross in his article at the occasion of Koch’s 60th birthday [75].

Koch’s scientific studies and his photogrammes were considered of high quality and deserve our admiration. However, Grassi had won the race on publishing the key to transmission of human parasites, though only just. Knowing that Koch refused contact with Grassi, it is clear that he never saw any of the preparations made by his Italian opponent.

Koch’s irritation about the role of Grassi before, during and after his major expedition to Italy and the Far East became an important stimulus to support the candidature of Ross for the Nobel Prize. Earlier presumptions [76] about the interfering role of Koch in this matter are now finally proven.

Koch surely did not abide with the concluding sentence of his Eastbourne lecture:

*In a few years the practical results of these [malaria control] experiments [among others by Ross in Sierra Leone] will be known to us, and then you may act on the good old saying, “Try all things; hold fast that which is good”.*

If Koch had been trying and holding fast the good things of Grassi’s work, the turmoil at the beginning of modern malaria research might have been eased. However, Grassi had not made things easy, either. In his beautiful book, he found fault with everything that his contenders in the race had discovered. Koch and Ross were not amused and joined forces in their lobbying and attempts to manipulate the opinion of peers and the Swedish Jury. The story illustrates how great men can be petty.

Had Koch bothered to write an overview of his own pioneering malaria studies, like Laveran (1881), Plehn (1890; 1901), Mannaberg (1893), Ziemann (1898), Grassi (1901), Schaudinn (1902), Ruge (1906), Ross (1910) and other malaria pioneers have done, he might have been remembered not only as a famous bacteriologist, but also as a great parasitologist and malaria-immunologist. Not all his ideas stand modern insights, i.e. his exclusive use of quinine to exterminate malaria. But it is remarkable that Koch’s major discovery on the development of immunity against malaria through repeated exposure became standard knowledge, whilst his name gradually faded away in reviews and handbooks on tropical medicine. May his role in early malaria research be recognised again.
